# Important missing information in Japan's risk management plans: a cross-sectional analysis after a decade of implementation

**DOI:** 10.3389/fmed.2026.1895400

**Published:** 2026-07-29

**Authors:** Natsuko Kameyama, Ayana Ide, Hideki Maeda

**Affiliations:** 1 Regulatory Science, Graduate School of Pharmaceutical Science, Meiji Pharmaceutical University, Tokyo, Japan; 2 CMIC Co., Ltd., Tokyo, Japan; 3 Regulatory Science, Faculty of Pharmacy, Meiji Pharmaceutical University, Tokyo, Japan

**Keywords:** drug labeling, Japanese pharmaceutical regulations, missing information, pharmacovigilance, risk management plan, safety concerns

## Abstract

**Introduction:**

More than 10 years have passed since Japan introduced risk management plans (RMPs), yet research focusing on important missing information (IMI) remains limited. This study aimed to characterize IMI, particularly in special populations.

**Methods:**

Based on publicly available information from the Pharmaceuticals and Medical Devices Agency, we analyzed 330 eligible RMPs as of May 2023 and summarized the number and classification of IMI items and associated activities.

**Results:**

Among 330 RMPs, 101 (30.6%) included 199 IMI items. Additional pharmacovigilance activities, additional risk minimization activities, and documentation in the package insert were identified for 69.3%, 25.1%, and 56.3% of the items, respectively. Special populations accounted for 62.8% of all IMI items, and their categorization aligned with International Council for Harmonisation guideline E2E. Additional pharmacovigilance activities were specified in the RMP for 64.0% of special population-related items, whereas the rate was significantly lower for older patients than for other special population categories (33.3%, *p* = 0.028). For long-term use (44 items, 22.1%), additional pharmacovigilance activities were specified in the RMP more frequently than other IMI categories (84.1%, *p* = 0.016), whereas additional risk minimization activities and documentation in the package insert were less frequent [11.4% (*p* = 0.018) and 13.6% (p < 0.001), respectively].

**Conclusion:**

Japan's approach to IMI has broadly aligned with international standards, particularly regarding special population-related IMI, while several operational issues remain. Strengthening additional pharmacovigilance activities for selected older patients, explicitly including long-term use as an example of IMI in Japanese RMP guidance, and defining IMI based on the need for additional activities might help enhance RMP management and support future regulatory refinement.

## 1 Introduction

A risk management plan (RMP) is a structured framework for risk management that builds upon and formalizes the concept of pharmacovigilance (PV) planning proposed in International Council for Harmonisation (ICH) guideline E2E (1). An RMP outlines risk management throughout the life cycle of a medicinal product from development to the post-marketing phase, and it consists of three components: safety concerns, PV activities, and risk minimization activities for each individual product (2, 3). In Japan, the “Guideline on Risk Management Plan” issued in 2012 applies to new drugs submitted for approval on or after April 1, 2013, with the RMP obligation imposed as a condition for marketing approval (4). In addition, following the 2025 amendment to the Act on Securing Quality, Efficacy and Safety of Products Including Pharmaceuticals and Medical Devices, RMP preparation and implementation is explicitly set to be mandated by law (5, 6). Thus, Japan's RMP system is currently in a period of institutional and operational transition.

More than 10 years have passed since the introduction of the RMP system in Japan, yet research focusing on RMPs remains limited. In particular, the actual operational status of important missing information (IMI) regarding safety concerns has not been fully elucidated. In Japan, IMI is defined as information “for which sufficient data are not available at the time of preparing the RMP and which is considered important for predicting the safety of the medical product in the post-marketing setting.” Examples include patient populations excluded from clinical trials but expected to be frequently treated in real-world clinical practice, for whom safety information is necessary (4).

This definition is based on the concept of “populations not studied or only studied to a limited degree in the pre-approval phase,” recommended in ICH E2E as candidate IMI, hereafter termed “special populations.” Examples include pediatric patients, older patients, pregnant or lactating women, patients with relevant comorbidities such as hepatic or renal disorders, patients with differences in disease severity compared with that studied in clinical trials, subpopulations carrying known and relevant genetic polymorphism, and patients of different racial and/or ethnic origins (1). Because safety concerns such as IMI can influence the benefit–risk balance of medicinal products or contribute to the occurrence or spread of public health harms (4), assessing the current status and operation of IMI is critically important for effective PV and risk management.

Regarding previous studies, Sawada et al. analyzed all active RMPs as of 2023, 10 years after the RMP system was introduced (7). We also investigated the characteristics of important potential risks at the same time point and highlighted issues related to RMP termination (8, 9). As Japan's RMP system is relatively new, conducting an evaluation 10 years after its implementation carries considerable significance. However, the characteristics of IMI and its relationship with associated activities have not been sufficiently examined. Therefore, this study investigated the characteristics of IMI in Japan, particularly that involving special populations, to enhance understanding of the current status of the RMP system and provide insights to support future system refinement.

## 2 Materials and methods

### 2.1 Data collection and processing

This cross-sectional study targeted RMPs that were active as of May 2023, provided that their content could be confirmed at the time of analysis in May 2024. RMPs that had been revised or removed by May 2024 were excluded because the publicly available information was insufficient to consistently verify that the RMP content available at the time of analysis corresponded to the content that was active as of May 2023. Information on RMPs was partially obtained from a database created by Sawada et al. (7) based on the list of active RMPs published on the Pharmaceuticals and Medical Devices Agency (PMDA) website (10). In that study, robotic process automation tools were used to extract product names, active ingredients, and safety concerns. Additional information on products covered by each RMP was obtained from the PMDA's “Information Search for Prescription Drugs” (11). From these sources, we obtained information on IMI, PV activities, and risk minimization activities and subsequently constructed an original database. Using this database, we organized and analyzed the number of IMI items per RMP, the number of IMI items by classification category, whether additional PV and additional risk minimization activities were specified in the RMP, and the status of documentation in the package insert (PI; Japanese product labeling) as routine risk minimization activities. Additional PV and additional risk minimization activities were assessed based on whether they were specified in the RMP at the time of analysis. The detailed implementation status of each activity, such as whether it was planned, ongoing, or completed, was not separately evaluated.

### 2.2 Classification of missing information

IMI items were classified into the following categories: safety in special populations, safety of long-term use, missing information related to dosage and administration, drug–drug interactions, missing information related to disease and symptoms, and others. In this study, “special populations” were defined based on the population groups described in ICH E2E as examples of missing information, namely populations not studied or only studied to a limited degree in the pre-approval phase (1). Items classified as special populations were further subclassified according to the population groups exemplified in ICH E2E. Regarding the other IMI categories, “safety of long-term use” was adopted from the examples of “anticipated utilization” included in the definition of IMI in EU-GVP Module V Rev. 2 (12). The remaining categories were selected because they represent classifications commonly used for IMI in Japan.

### 2.3 Statistical analysis

This study was conducted per the Strengthening the Reporting of Observational Studies in Epidemiology (STROBE) reporting guidelines for cross-sectional studies (13). All analyses were performed using R (version 4.5.1; R Foundation for Statistical Computing, Vienna, Austria). Categorical variables were compared using Fisher's exact test based on 2 × 2 contingency tables comparing each category or subcategory of interest with all other categories or subcategories combined, with two-sided *p* < 0.05 considered statistically significant. For these comparisons, odds ratios (ORs) and 95% confidence intervals (CIs) were reported.

## 3 Results

### 3.1 Overview and classification of IMI

**Figure 1:**
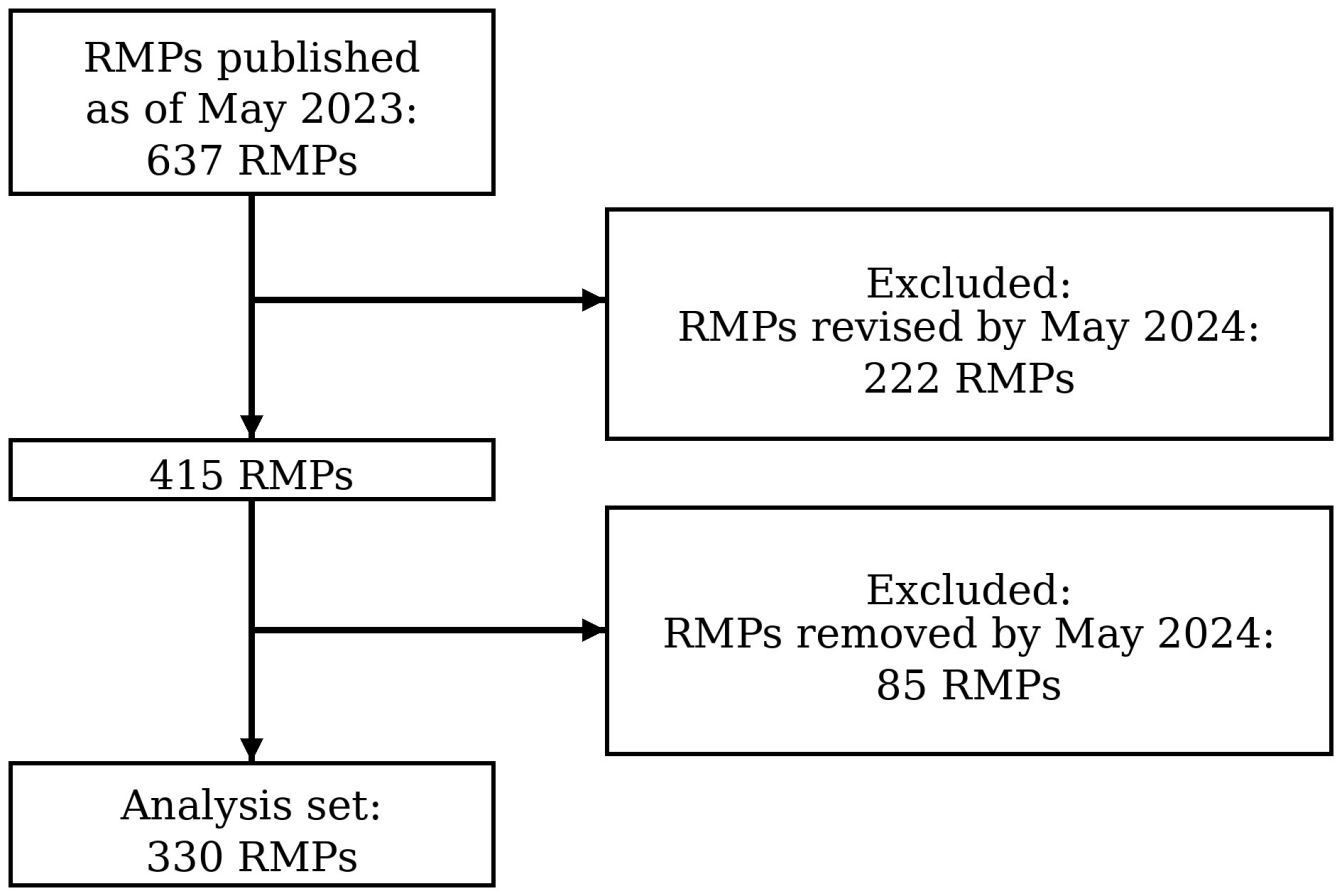
Inclusion and exclusion criteria. RMPs published as of May 2023 were screened. RMPs revised or removed by May 2024 were excluded because it could not be consistently confirmed that the RMP content available at the time of analysis corresponded to the content that was active as of May 2023, resulting in an analysis set of 330 RMPs.

**Figure 2:**
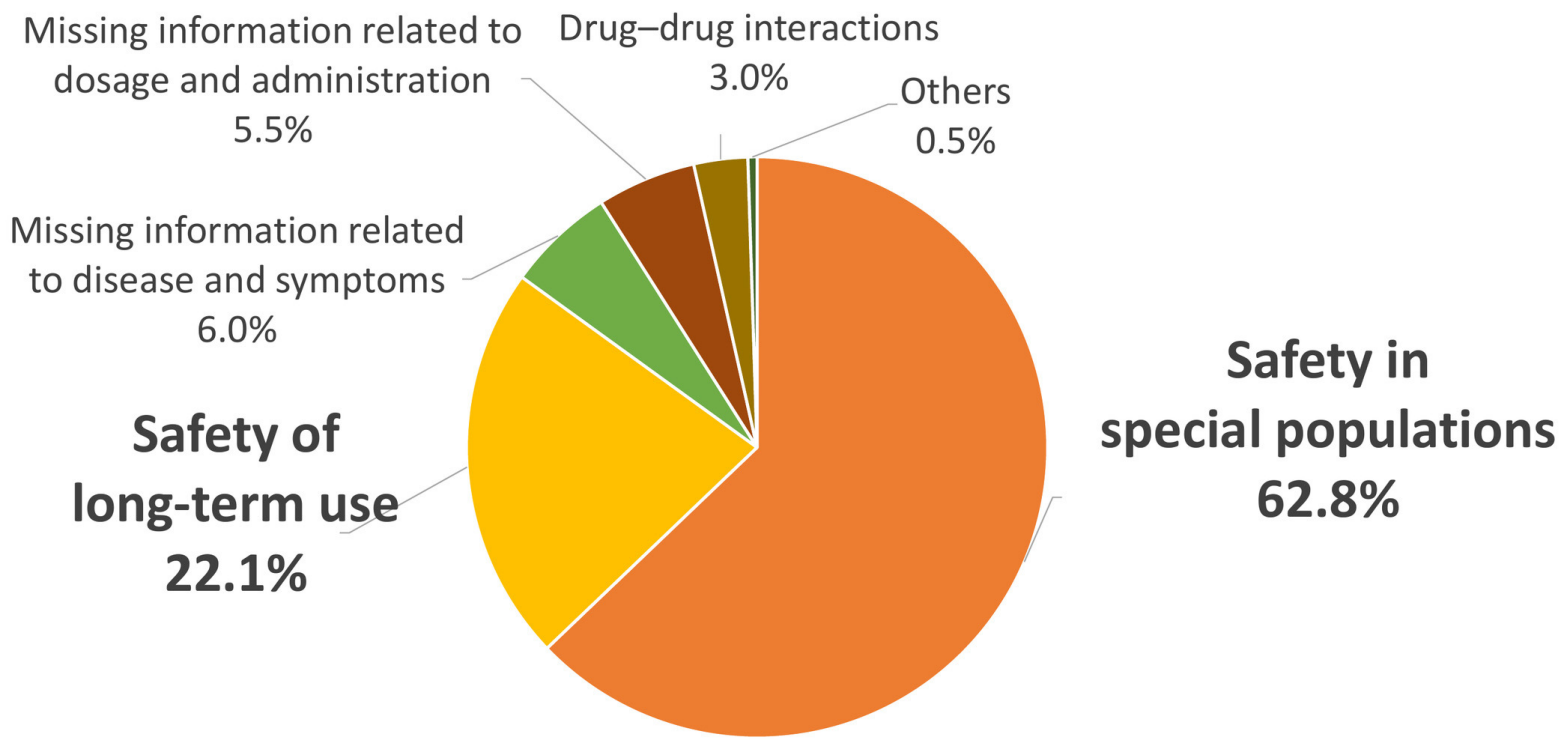
Distribution of important missing information categories. The 199 important missing information items identified in the analyzed RMPs were categorized by type.

Among the 637 RMPs publicly available as of May 2023, 222 RMPs that were revised and 85 RMPs that were deleted between June 2023 and May 2024 were excluded because it could not be consistently confirmed using publicly available information that the RMP content available at the time of analysis corresponded to the content that was active as of May 2023, leaving 330 RMPs for analysis (Figure 1). To assess the potential impact of selection bias related to the exclusion of revised or removed RMPs, we compared basic characteristics between the included and excluded RMPs. Therapeutic category (14) and initial RMP submission year were summarized in Supplementary Table S1. The distributions were broadly similar between the included and excluded RMPs, although the excluded RMPs included slightly higher proportions of antineoplastic agents, agents affecting the central nervous system, and RMPs initially submitted in 2021–2023. Among the 330 RMPs included in the analysis, 101 products (30.6%) had at least one IMI item, and nearly half of these had only one IMI item (Table 1). These 101 products contained a total of 199 IMI items. Of these, safety in special populations was the most populous category, accounting for 125 items (62.8%), followed by safety of long-term use [44 items (22.1%), Table 2, Figure 2]. Among the 199 IMI items, additional PV activities, additional risk minimization activities, and documentation in the PI were identified for 138 (69.3%), 50 (25.1%), and 112 items (56.3%), respectively (Table 3). To further characterize additional PV activities, we summarized their types among IMI items for which the type of additional PV activity specified in the RMP could be identified at the time of the supplementary analysis. Use-results surveys comprised the most common type of additional PV activity, whereas other types were less frequently used (Supplementary Table S2). As special populations accounted for most IMI items, further analyses focused on this category.

**Table 1:** Distribution of important missing information among risk management plans.

Risk management plan classification		*N*
Risk management plans		**330**
No missing information		229
With missing information		101
Number of missing information items	1	48
	2	21
	3	22
	4	7
	5	3

**Table 2:** Characteristics of important missing information and special populations.

Important missing information category	*N*	%
**Important missing information**	**199**	100.0
Safety in special populations	125	62.8
Patients with hepatic disorders	*24*	*19.2*
Patients with renal disorders	*23*	*18.4*
Pediatric patients	*19*	*15.2*
Pregnant women	*14*	*11.2*
Older patients	*12*	*9.6*
Patients with different severities or subtypes of the underlying disease	*8*	*6.4*
Patients of different racial and/or ethnic origins	*8*	*6.4*
Other specific patient populations	*17*	*13.6*
Safety of long-term use	44	22.1
Missing information related to disease and symptoms	12	6.0
Missing information related to dosage and administration	11	5.5
Drug–drug interactions	6	3.0
Others	1	0.5

Italic values indicate special population subcategories.

**Table 3:** Additional activities and package insert documentation for important missing information.

Important missing information category	Additional pharmacovigilance activities					Additional risk minimization activities				Documentation in the package insert			
	*N*	*n*	%	*p*	OR (95% CI)	*n*	%	*p*	OR (95% CI)	*n*	%	*p*	OR (95% CI)
**Important missing information**	**199**	**138**	**69.3**			**50**	**25.1**			**112**	**56.3**		
Safety in special populations	**125**	**80**	**64.0**	**0.039**	**0.49 (0.24–0.99)**	35	28.0	0.242	1.53 (0.74–3.29)	**92**	**73.6**	**< 0.001**	**7.44 (3.76–15.23)**
Patients with hepatic disorders	*24*	*14*	*58.3*	*0.637*	*0.74 (0.27–2.08)*	*9*	*37.5*	*0.312*	*1.72 (0.59–4.82)*	*19*	*79.2*	*0.611*	*1.45 (0.46–5.47)*
Patients with renal disorders	*23*	*13*	*56.5*	*0.473*	*0.68 (0.25–1.93)*	*6*	*26.1*	*1.000*	*0.89 (0.26–2.67)*	***22***	***95.7***	***0.008***	***9.94 (1.47–427.00)***
Pediatric patients	*19*	*12*	*63.2*	*1.000*	*0.96 (0.32–3.13)*	*4*	*21.1*	*0.585*	*0.65 (0.14–2.25)*	*13*	*68.4*	*0.580*	*0.74 (0.23–2.62)*
Pregnant women	*14*	*9*	*64.3*	*1.000*	*1.01 (0.28–4.13)*	*1*	*7.1*	*0.110*	*0.18 (0.00–1.26)*	***14***	***100.0***	***0.020***	***∞(1.30–∞)***
Older patients	***12***	***4***	***33.3***	***0.028***	***0.25 (0.05–0.99)***	*5*	*41.7*	*0.314*	*1.96 (0.46–7.83)*	*10*	*83.3*	*0.515*	*1.88 (0.37–18.62)*
Patients with different severities or subtypes of the underlying disease	*8*	*6*	*75.0*	*0.710*	*1.74 (0.29–18.32)*	*2*	*25.0*	*1.000*	*0.85 (0.08–5.07)*	*6*	*75.0*	*1.000*	*1.08 (0.18–11.50)*
Patients of different racial and/or ethnic origins	***8***	***8***	***100.0***	***0.0498***	***∞(1.00–∞)***	*0*	*0.0*	*0.104*	*0.00 (0.00–1.46)*	***0***	***0.0***	*** < 0.001***	***0.00 (0.00–0.18)***
Other specific patient populations	*17*	*14*	*82.4*			*8*	*47.1*			*8*	*47.1*		
Safety of long-term use	**44**	**37**	**84.1**	**0.016**	**2.81 (1.14–7.99)**	**5**	**11.4**	**0.018**	**0.31 (0.09–0.87)**	**6**	**13.6**	**< 0.001**	**0.07 (0.02–0.19)**
Missing information related to disease and symptoms	12	10	83.3	0.351	2.30 (0.47–22.21)	2	16.7	0.73	0.58 (0.06–2.87)	**2**	**16.7**	**0.006**	**0.14 (0.01–0.69)**
Missing information related to dosage and administration	11	8	72.7	1.000	1.19 (0.27–7.21)	4	36.4	0.473	1.76 (0.36–7.29)	7	63.6	0.75	1.38 (0.34–6.66)
Drug–drug interactions	6	2	33.3	0.073	0.21 (0.02–1.52)	3	50.0	0.168	3.09 (0.40–23.82)	5	83.3	0.234	4.00 (0.44–192.07)
Others	1	1	100.0			1	100.0			0	0.0		

OR, odds ratio; CI, confidence interval. ORs compare each category or subcategory of interest with all other categories or subcategories combined. *P* values were calculated using Fisher's exact test. Boundary values of 0 and infinity for ORs and 95% CIs reflect the complete absence and presence of the corresponding outcome in the subgroup, respectively. Italic values indicate special population subcategories.

### 3.2 Characteristics and activities of special populations

**Figure 3:**
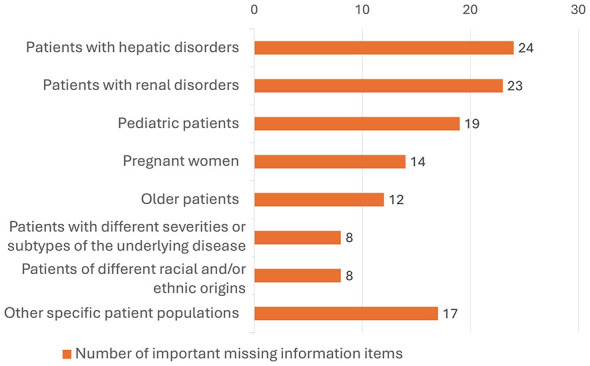
Number of important missing information items by special population category. The 125 important missing information items classified as safety in special populations were summarized according to subpopulation category. RMP, risk management plan.

Among the 125 IMI items targeting special populations, 24 (19.2%), 23 (18.4%), 19 (15.2%), 14 (11.2%), and 12 (9.6%) concerned patients with hepatic disorders, patients with renal disorders, pediatric patients, pregnant women, and older patients, respectively. In addition, eight items (6.4%) concerned patients with different severities or subtypes of the underlying disease, whereas eight other items (6.4%) concerned patients of different racial and/or ethnic origins. Because few subpopulations were characterized by genetic polymorphisms known or predicted to be associated with safety concerns, these populations were aggregated, together with patients with comorbidities other than hepatic or renal disorders and populations defined by differences in sex, age, or body weight, into the category “other specific patient populations” (Table 2, Figure 3).

For special populations, additional PV activities were specified in the RMP for 64.0% of the items, a significantly lower proportion than those for other IMI categories (OR = 0.49, 95% CI = 0.24–0.99, *p* = 0.039). At the level of individual subgroups, the proportion was significantly higher for patients of different racial and/or ethnic origins (100.0%; OR = ∞, 95% CI = 1.00–∞, *p* = 0.0498), whereas it was significantly lower for older patients (33.3%; OR = 0.25, 95% CI = 0.05–0.99, *p* = 0.028). Documentation in the PI was observed for 73.6% of IMI items related to special populations, a significantly higher rate than that observed for other IMI categories (OR = 7.44, 95% CI = 3.76–15.23, *p* < 0.001). In particular, the proportion was significantly higher for pregnant women (100.0%; OR = ∞, 95% CI = 1.30–∞, *p* = 0.020) and patients with renal disorders (95.7%; OR = 9.94, 95% CI = 1.47–427.00, *p* = 0.008), whereas it remained extremely low for patients of different racial and/or ethnic origins (0.0%; OR = 0.00, 95% CI = 0.00–0.18, *p* < 0.001; Table 3).

### 3.3 Activities of long-term safety concerns

Safety of long-term use was the second most frequent IMI category after special populations. For this category, additional PV activities were specified in the RMP for 84.1% of items, significantly exceeding the rates for other IMI categories (OR = 2.81, 95% CI = 1.14–7.99, *p* = 0.016). Conversely, additional risk minimization activities and documentation in the PI were identified in significantly lower proportions, at 11.4% (OR = 0.31, 95% CI = 0.09–0.87, *p* = 0.018) and 13.6% (OR = 0.07, 95% CI = 0.02–0.19, *p* < 0.001), respectively (Table 3).

## 4 Discussion

The findings of this study indicated that only one-third of medicinal products had IMI specified in their RMPs, and even among those with IMI, nearly half had only a single IMI item. This trend is consistent with previous findings that the mean number of IMI items per product was 0.65, considerably lower than the mean numbers of important identified risks (4.92) and important potential risks (2.72) (7). Studies comparing Japan and the EU have also reported that the number of IMI items specified in Japan is relatively low (15–17). These findings indicate that one contributing factor is that, in Japan, IMI has traditionally been limited to patient populations within the approved indication (15).

Meanwhile, additional PV activities were specified in the RMP for 69.3% of IMI items, a proportion exceeding that observed for important potential risks (41.8%; 323 of 772 items, data not shown). In 2019, regulatory authorities emphasized that PV plans should be scientifically justified and stated that additional studies should not be uniformly required solely because of small clinical trial sample sizes or limited information in specific populations (18). The usefulness of comprehensive data collection traditionally conducted in general drug use results surveys has also been questioned (19), and increasing importance has been placed on designing scientifically justified PV plans using diverse methodologies, including database studies when appropriate data sources and pharmacoepidemiologic methods are available (20, 21). Taken together, these developments suggest that, although the number of IMI items is relatively small, the items designated as IMI might have been focused on those warranting additional PV activities, consistent with the increasing emphasis on scientifically justified PV planning.

Special population-related IMI was found to be aligned with the classifications recommended in ICH E2E, indicating that appropriate population groups were captured in accordance with international standards. This finding should be interpreted in light of the regulatory concept of missing information, which forms the basis for IMI designation in RMPs. According to ICH E2E and the Japanese RMP guidance (1, 4), missing information includes clinically important gaps in safety knowledge, such as limited experience in populations not adequately studied in the pre-approval phase or information needed to predict post-marketing safety in patient populations excluded from clinical trials but expected to be treated in real-world practice. Thus, the high rate of special population-related IMI suggests that limited pre-approval evidence in these populations, which are often difficult to adequately study in clinical trials, is an important driver of IMI designation in Japanese RMPs. Additional PV activities were also specified in the RMP for 55%−100% of items across most subcategories, suggesting that the current operational practices are largely appropriate. The high rate of PI documentation for special population-related IMI suggests that these items are often addressed through routine risk minimization in the PI, particularly through Section 9 [“Precautions Concerning Patients with Specific Backgrounds” (22)].

However, among the special populations, additional PV activities were specified in the RMP for only 33.3% of older patient-related IMI items. One possible explanation for this lower proportion is that sponsors might consider routine PV activities, such as spontaneous reporting, sufficient when older patients are expected to be included to some extent among the patients treated in the post-marketing setting. In such cases, additional PV activities specifically targeting older patients might not be considered necessary, even when older patients are designated as an IMI. This could partly explain the observed discrepancy between the designation of older patients as IMI and the limited specification of additional PV activities for this population. Nevertheless, the limited enrollment of older patients in clinical trials and the frequent lack of clinically meaningful detail in the PI raise concerns regarding the usefulness of the available information in real-world clinical practice (23). Moreover, from the perspective of the ICH E2E concept, the designation of older patients as an IMI should reflect not merely the absence of information but a clinically relevant uncertainty regarding post-marketing safety. Therefore, rather than relying solely on broad information collection through routine PV activities, it might be necessary to establish specific research questions, proactively collect and evaluate relevant safety information through additional PV activities, and incorporate the resulting evidence into the PI in a more clinically meaningful manner.

Beyond special populations, other types of IMI also warrant attention. Among IMI items other than those related to special populations, safety of long-term use was frequently observed. This finding might reflect the limited duration of pre-approval clinical trials and the resulting uncertainty regarding long-term safety in real-world use. Although the EU-GVP lists anticipated utilization (e.g., long-term use) as an example of IMI, the Japanese RMP guideline provides examples only for safety in specific patient populations (4, 12). As discussions on shortening treatment duration in clinical trials progress (24, 25), the importance of evaluating the safety of long-term use in the post-marketing setting is expected to increase. Therefore, it would be desirable for the Japanese RMP guideline to explicitly list long-term use as an example of IMI.

From a regulatory perspective, the fact that no additional PV activities were specified in the RMP for approximately 30% of IMI items raises the question of whether information for which such activities are not necessary should actually be included as safety concerns. In this context, differences between the Japanese and EU approaches to defining missing information are also relevant. In the EU, the category is simply labeled “missing information,” yet only uncertainties with a clear scientific rationale are included. The absence of data (e.g., exclusion of a population from clinical studies) is not sufficient on its own (12, 26). By contrast, Japan adopts the ICH term “important missing information” verbatim but applies less stringent criteria at the designation stage. Although scientific justification is required when determining additional PV activities (18), it is not applied as rigorously when defining IMI, resulting in a relatively broad operational scope. This might also explain why some IMI items remain designated even when the need for additional activities is unclear. This issue could become more important as Japan transitions to a system in which RMP preparation and implementation are explicitly mandated by law. If broadly defined IMI items remain listed in RMPs without sufficient operational clarity regarding their reassessment, updating, or removal, they might increase compliance and operational burdens for marketing authorization holders and reduce the clarity of RMP management.

To address this issue, Narukawa (27) has proposed a dynamic operational framework in which only items requiring additional activities are designated as safety concerns and subsequently updated as those activities progress or are completed. We agree with this perspective and consider that the designation of IMI items as safety concerns should be reviewed according to whether additional activities are warranted.

In addition, a recent study that tracked post-approval changes in RMPs reported that many safety concerns remained listed in RMPs for up to 8 years after approval (28). This finding suggests that the current system does not readily facilitate the organization or removal of items. Accordingly, institutional improvements are needed to ensure that RMPs are managed dynamically and that IMI items no longer requiring additional activities are reliably removed.

This study had several limitations. First, this was a cross-sectional analysis based on RMPs publicly available in 2023, and differences in the number of years elapsed since approval for each product might have influenced the findings. Second, because RMPs revised or removed by May 2024 were excluded, the analysis did not include all RMPs active as of May 2023. Although their exclusion was necessary to ensure the consistency and verifiability of the analyzed RMP content using publicly available information, this might have introduced selection bias. In the supplementary comparison, the distributions of therapeutic category and initial RMP submission year were broadly similar between the included and excluded RMPs, although some differences were observed. Thus, although the supplementary comparison did not suggest a substantial imbalance in these basic characteristics, the potential impact of excluding revised or removed RMPs should be considered when interpreting the findings. Future longitudinal studies, including revised and removed RMPs, are needed to more comprehensively evaluate how IMI and associated activities change over time. Third, this study assessed whether additional PV activities were specified in the RMP but did not determine whether each activity was planned, ongoing, or completed at the time of analysis, as the RMP text does not always clearly distinguish these statuses. Therefore, the reported proportions should be interpreted as the proportions of IMI items for which additional PV activities were specified in the RMP, rather than as confirmed proportions of activities actively ongoing at the time of analysis. Fourth, multiple regulatory changes occurred during the study period, which could have affected the results. Future longitudinal studies tracking changes in safety concerns and their associated activities over time will be essential to more accurately capture how IMI evolves throughout the life cycle of each medicinal product. Despite these methodological limitations, this study offers an academic and practical contribution by analyzing IMI across 330 verifiable RMPs and offering a detailed assessment of IMI within Japan's RMP system a decade after its implementation.

## 5 Conclusions

Japan's approach to IMI was broadly consistent with international standards such as ICH E2E while also exhibiting unique characteristics. To further strengthen the RMP system, the following improvements are warranted: enhancing safety information for selected older patients through more targeted post-marketing PV activities; explicitly incorporating long-term use as an example of IMI in the Japanese RMP guideline; and adopting a more dynamic framework in which IMI items are designated according to the need for additional activities and are reassessed when warranted. These measures might help improve the clarity and effectiveness of RMP management and support future regulatory refinement and international harmonization.

## Data Availability

The raw data supporting the conclusions of this article will be made available by the authors, without undue reservation.
