# Supplementary material for: Important missing information in Japan's risk management plans: a cross-sectional analysis after a decade of implementation
Source: Front Med (Lausanne). 2026 Jul 29;13:1895400. doi: 10.3389/fmed.2026.1895400 (PMC13461612; doi:10.3389/fmed.2026.1895400)
Supplement: Supplementary file 1 [file Table_1.docx]

Supplementary Material

# 1 Supplementary Tables

**Supplementary Table S1.** Characteristics of included and excluded RMPs.

| Characteristic | | Included RMPs  (N=330), n (%) | Excluded RMPs  (N=307), n (%) |
| --- | --- | --- | --- |
| Therapeutic category | |  |  |
|  | Other agents affecting metabolism | 64 (19.4) | 50 (16.3) |
|  | Antineoplastic agents | 47 (14.2) | 72 (23.5) |
|  | Agents affecting central nervous system | 33 (10.0) | 52 (16.9) |
|  | Biological preparations | 26 (7.9) | 26 (8.5) |
|  | Agents affecting digestive organs | 22 (6.7) | 7 (2.3) |
|  | Hormone preparations, including antihormone preparations | 20 (6.1) | 13 (4.2) |
|  | Chemotherapeutics | 20 (6.1) | 19 (6.2) |
|  | Cardiovascular agents | 18 (5.5) | 16 (5.2) |
|  | Antibiotic preparations | 10 (3.0) | 8 (2.6) |
|  | Antiallergic agents | 9 (2.7) | 9 (2.9) |
|  | Agents affecting sensory organs | 9 (2.7) | 6 (2.0) |
|  | Agents affecting respiratory organs | 9 (2.7) | 6 (2.0) |
|  | Dermatologic preparations | 9 (2.7) | 2 (0.7) |
|  | Agents affecting peripheral nervous system | 5 (1.5) | 2 (0.7) |
|  | Diagnostic agents, excluding in vitro diagnostics | 5 (1.5) | 0 (0.0) |
|  | Agents affecting blood and body fluids | 3 (0.9) | 6 (2.0) |
|  | Tonics | 3 (0.9) | 1 (0.3) |
|  | Alkaloid narcotics | 3 (0.9) | 1 (0.3) |
|  | Parasiticides | 3 (0.9) | 0 (0.0) |
|  | Radioactive pharmaceuticals | 2 (0.6) | 2 (0.7) |
|  | Nonalkaloid narcotics | 2 (0.6) | 1 (0.3) |
|  | Agents for urogenital organs and anus | 1 (0.3) | 2 (0.7) |
|  | Other agents not mainly intended for therapeutic purpose | 1 (0.3) | 1 (0.3) |
|  | Other agents affecting nervous system and sensory organs | 1 (0.3) | 0 (0.0) |
|  | Dental and oral preparations | 1 (0.3) | 0 (0.0) |
|  | Other agents affecting individual organs | 1 (0.3) | 0 (0.0) |
|  | Products with multiple therapeutic categories | 3 (0.9) | 5 (1.6) |
| Initial RMP submission year | |  |  |
|  | 2013–2017 | 107 (32.4) | 81 (26.4) |
|  | 2018–2020 | 115 (34.8) | 103 (33.6) |
|  | 2021–2023 | 108 (32.7) | 123 (40.1) |

**Note:** Percentages were calculated using each column total as the denominator. Products with multiple therapeutic categories were presented separately rather than being assigned to a single therapeutic category. RMP, risk management plan.

**Supplementary Table S2.** Types of additional pharmacovigilance activities specified in the RMPs

| Category | IMI items with additional PV activities, N | Use-results survey, n (%) | Post-marketing database study, n (%) | Post-marketing clinical study, n (%) | Other PV activities, n (%) |
| --- | --- | --- | --- | --- | --- |
| All IMI items | 107 | 103 (96.3) | 2 (1.9) | 5 (4.7) | 5 (4.7) |
| Safety in special populations | 61 | 60 (98.4) | 0 (0.0) | 1 (1.6) | 2 (3.3) |
| Safety of long-term use | 32 | 32 (100.0) | 0 (0.0) | 4 (12.5) | 1 (3.1) |
| Missing information related to disease and symptoms | 5 | 3 (60.0) | 1 (20.0) | 0 (0.0) | 1 (20.0) |
| Missing information related to dosage and administration | 6 | 5 (83.3) | 1 (16.7) | 0 (0.0) | 0 (0.0) |
| Drug–drug interactions | 2 | 2 (100.0) | 0 (0.0) | 0 (0.0) | 1 (50.0) |
| Others | 1 | 1 (100.0) | 0 (0.0) | 0 (0.0) | 0 (0.0) |

**Note:** This supplementary analysis included IMI items for which the type of additional PV activity specified in the RMP could be identified at the time of the supplementary analysis. Percentages were calculated using the number of IMI items with additional PV activities in each category as the denominator, and the summed percentages might exceed 100% because multiple activities could be specified for a single IMI item. Use-results surveys included general, specified, comparative, and unspecified surveys. Other PV activities comprised registry-based surveys and early post-marketing phase vigilance. IMI, important missing information; PV, pharmacovigilance; RMP, risk management plan.
